# ARL3 Enhances ERα Stability via USP10 Deubiquitination to Promote Endocrine Resistance and Drive Mitochondrial Metabolic Reprogramming in HR+ Breast Cancer

**DOI:** 10.1002/advs.202509769

**Published:** 2025-10-05

**Authors:** Han Li, Yang Liu, Zehao Cai, Kang Li, Shun Gao, Ailin Lan, Dan Shu, Kuan He, Xin Liu, Yang Peng, Shipeng Guo, Haochen Yu, Aishun Jin, Meiying Shen, Shengchun Liu

**Affiliations:** ^1^ Department of Breast and Thyroid Surgery Chongqing Key Laboratory of Molecular Oncology and Epigenetics The First Affiliated Hospital of Chongqing Medical University Chongqing 400016 China; ^2^ Department of Immunology School of Basic Medical Sciences Chongqing Medical University Chongqing 400010 China; ^3^ Chongqing Key Laboratory of Tumor Immune Regulation and Immune Intervention Chongqing 400010 China; ^4^ Department of Critical Care Medicine The First Affiliated Hospital of Chongqing Medical University Chongqing 400016 China

**Keywords:** ARL3, Endocrine therapy, ESR1, HR+ breast cancer, USP10

## Abstract

The molecular mechanisms of estrogen receptor α (ERα)‐positive breast carcinogenesis and endocrine resistance remain unclear. This study identifies ADP‐ribosylation factor‐like protein 3 (ARL3) as a key oncogenic regulator overexpressed in ERα‐positive breast cancer cells and tissues. Mechanistically, ARL3 stabilizes ERα as a novel chaperone via direct binding, enhancing ESR1‐driven transcription and cell proliferation. Genetic ablation of ARL3 induces ERα ubiquitination‐dependent degradation, activating mTOR/AMPK pathways and causing mitophagy/mitochondrial dysfunction. ARL3 maintains ERα stability by upregulating USP10, which removes K48/K63‐linked polyubiquitin chains from ERα at the K252 site. In preclinical models, the small‐molecule inhibitor A‐1331852 (targeting ARL3) potently suppresses ERα‐positive tumor growth and synergizes with endocrine therapies. These findings establish ARL3 as a critical regulator of ERα homeostasis via USP10, highlighting its dual role as a biomarker and ARL3‐targeted therapeutic for ERα‐positive breast cancer.

## Graphical abstract

This study identifies ADP‐ribosylation factor‐like protein 3 (ARL3) as a novel chaperone that stabilizes estrogen receptor α (ERα) in breast cancer. ARL3 promotes tumor growth by recruiting USP10 to remove ubiquitin chains from ERα, preventing its degradation. Targeting ARL3 with a small‐molecule inhibitor suppresses tumor growth and synergizes with endocrine therapy, highlighting its therapeutic potential.

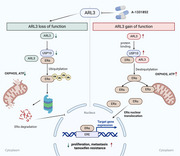

## 1 Introduction

Breast cancer represents the most prevalent malignancy among women worldwide,^[^ ^1^, ^2^ ^]^ with the hormone receptor‐positive (HR+) subtype constituting the predominant molecular subtype.^[^ ^3^, ^4^ ^]^ Estrogen receptor α (ERα) plays a pivotal role in normal mammary physiology and HR+ breast carcinogenesis.^[^ ^5^ ^]^ While endocrine therapies targeting ERα signaling pathways (e.g., tamoxifen) remain cornerstone treatments^[^ ^6^, ^7^ ^]^ ≈30–40% of patients develop metastatic recurrence due to acquired resistance.^[^ ^3^ ^]^ Current mechanistic understanding of endocrine resistance encompasses multiple molecular pathways, such as ERα signaling aberrations, somatic mutations, epigenetic reprogramming, and tumor microenvironmental adaptations, with additional pathways remaining under active investigation.^[^ ^8^ ^]^

The functional plasticity of ERα is dictated by its structural domains and post‐translational modifications (PTMs).^[^ ^9^, ^10^ ^]^ This nuclear receptor contains distinct functional modules—the N‐terminal activation function 1 (AF1) domain undergoing phosphorylation‐mediated activation by mitogenic kinases, the central DNA‐binding domain (DBD) facilitating estrogen response element (ERE) recognition, and the C‐terminal ligand‐binding domain (LBD) harboring activation function 2 (AF2) to coordinate nuclear translocation and co‐regulator recruitment.^[^ ^11^, ^12^ ^]^ Furthermore, ERα overexpression drives oncogenesis via upregulation of cell cycle regulators (e.g., cyclin D1) and proto‐oncogenes (e.g., c‐Myc).^[^ ^13^, ^14^ ^]^ ERα undergoes dynamic PTM regulation, with ubiquitination playing dual roles in its functional modulation.^[^ ^11^, ^15^, ^16^ ^]^ The ubiquitin‐proteasome system (UPS) exerts paradoxical regulatory effects through the coordinated actions of E3 ligases and deubiquitinating enzymes (DUBs). It has been shown that the E3 ligases RNF181 and RNF31 can stabilize estrogen receptor α (ERα) and influence breast cancer progression.^[^ ^11^, ^16^ ^]^ Meanwhile, other E3 ligases such as TRIM56 promote K63–linked polyubiquitination, which stabilizes ERα and fosters tamoxifen resistance.^[^ ^17^ ^]^ Conversely, counter–regulatory DUBs, including OTUD7B and USP36, regulate ERα degradation by editing ubiquitin chains.^[^ ^18^, ^19^ ^]^ These findings highlight the critical need to elucidate ERα regulatory networks, particularly at post‐translational levels.

Emerging evidence underscores metabolic plasticity as a pivotal determinant of chemotherapeutic susceptibility, with therapy‐resistant malignancies dynamically rewiring energy metabolism to evade cytotoxic stress.^[^ ^20^ ^]^ In tamoxifen‐resistant (TAM‐R) breast cancer, divergent metabolic adaptations coexist with glucose deprivation‐induced HK2 overexpression, which suppresses mTORC1 activity to activate pro‐survival autophagy through glycolytic flux escalation,^[^ ^21^, ^22^ ^]^ while paradoxically, mitochondrial OXPHOS hyperactivation defines a subset of TAM‐R clones susceptible to OXPHOS‐targeted resensitization strategies.^[^ ^3^ ^]^ Nevertheless, the metabolic consequences of the ERα–regulated pathway remain unclear. Earlier investigations have suggested that mutations in the ligand‐binding domain (LBD) of ESR1 result in a more aggressive phenotype. This phenotype is linked to a distinct gene signature and metabolic profile.^[^ ^23^ ^]^ Therefore, researching ER‐mediated metabolic pathways is essential for unravelling breast cancer mechanisms and devising better therapies.

To systematically identify novel ERα regulators, we performed integrative analysis of endocrine resistance‐associated transcriptomic datasets from the Gene Expression Omnibus (GEO). We identified ARL3 (ADP‐ribosylation factor‐like protein 3), an understudied member of the RAS GTPase superfamily, as a key candidate. ARL3 contains conserved GTPase domains (G1–G5) that mediate nucleotide‐dependent conformational switching.^[^ ^24^, ^25^ ^]^ ARF family proteins are known regulators of various tumors,^[^ ^26^, ^27^ ^]^ but ARL3's role in breast cancer remains unexplored. Herein, we demonstrate that ARL3 promotes breast cancer progression and tamoxifen resistance through coordinating dual regulatory mechanisms, stabilizing ERα through USP10‐mediated deubiquitination of both K48‐ and K63‐linked chains and modulating ERα‐mTOR/AMPK crosstalk to enhance mitochondrial oxidative phosphorylation (OXPHOS). Overall, our findings revealed that the ARL3/USP10/ERα complex plays a pivotal role in driving endocrine resistance. This complex has been pinpointed as a promising therapeutic target for HR+ breast cancer, highlighting its potential significance in advancing treatment strategies for this specific subtype of breast cancer.

## 2 Results

### 2.1 ARL3 Overexpression in Luminal Breast Cancer Associates with Adverse Prognosis

**Figure 1:**
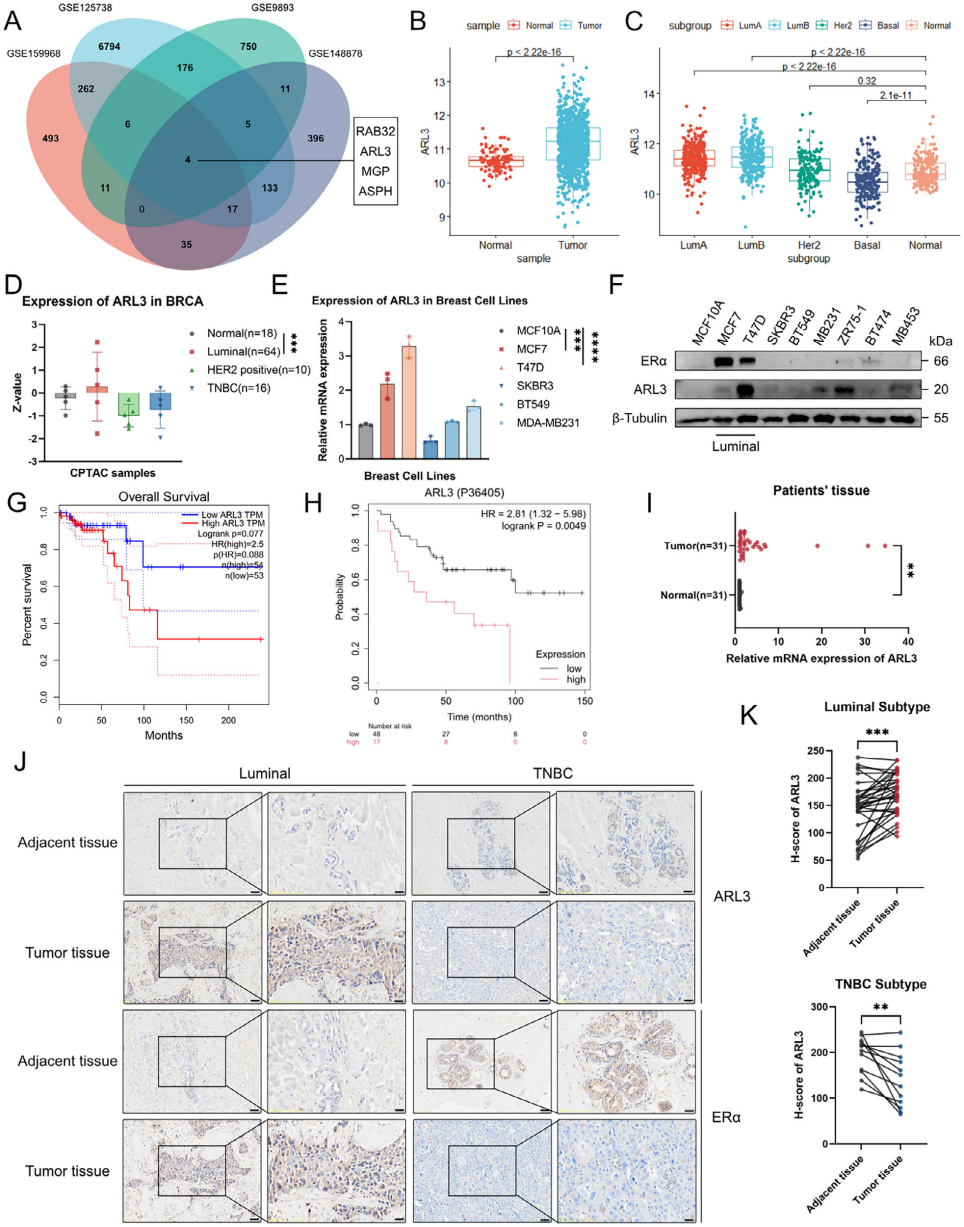
ARL3 correlates with the luminal subtype and predicts poor prognosis in breast cancer. A) Venn diagram of shared differentially expressed genes among tamoxifen‐resistant MCF7 (GSE159968 and GSE148878 datasets), T47D (GSE125738 dataset), and endocrine therapy‐relapsed patients (GSE9893 dataset). B‐D) Comparative ARL3 mRNA (TCGA dataset) and protein (CPTAC dataset) expression profiles in breast cancer tissues. E,F) ARL3 mRNA (E) and protein expression levels (F) across breast cancer cell lines. G,H) Overall survival analysis (GEPIA database) (G) and Kaplan‐Meier survival curves based on ARL3 protein expression levels (KM Plot database) (H). I) Quantitative PCR analysis of ARL3 expression in 31 paired breast cancer tissues and adjacent normal tissues (^**^ *p* < 0.01, paired two‐tailed Student's *t*‐test). J) Representative immunohistochemical staining of ARL3 and ESR1 in human breast cancer tissue and matched adjacent normal tissue. Scare bars, 20 µm. K) Quantitative analysis of ARL3‐positive cells in luminal breast cancer (*n* = 34, ^***^ *p* < 0.001) and triple‐negative breast cancer (TNBC) (*n* = 12, ^**^ *p* < 0.01) tissues versus adjacent normal tissues (paired two‐tailed Student's *t*‐test).

To identify regulators of endocrine resistance in breast cancer, we integrated four GEO datasets comprising tamoxifen‐resistant cell line models and clinical samples from neoadjuvant therapy nonresponders, identifying ARL3 as a top‐ranked candidate through cross‐dataset analysis of differentially expressed genes (FDR <0.05, fold‐change >1, **Figure** **1**A). Pan‐cancer expression analysis of TCGA‐BRCA (*n* = 1,085) and CPTAC (*n* = 122) datasets identified ARL3 as the only gene consistently overexpressed in tumor versus matched normal tissues after excluding other genes without significant prognostic associations, with pronounced enrichment in luminal A/B subtypes (Figure 1B–D; Figure S1A–F, Supporting Information). Consistent with clinical observations, ARL3 mRNA and protein levels were selectively elevated in ERα‐positive breast cancer cell lines compared to normal mammary epithelial cells (Figure 1E,F). Prognostic meta‐analysis using GEPIA and KM plotter demonstrated that elevated ARL3 expression correlates with significantly shorter overall survival in pan‐breast cancer cohorts (Figure 1G,H), with particularly adverse outcomes in luminal breast cancer patients (Figure S1G, Supporting Information). ARL3 mRNA expression was also significantly elevated in tumor tissues compared to adjacent normal counterparts (Figure 1I), consistent with its subtype‐specific overexpression in luminal breast cancer. Immunohistochemical profiling also revealed distinct subcellular localization patterns; luminal carcinomas exhibited predominant cytoplasmic ARL3 accumulation, contrasting with minimal expression in triple‐negative tumors (Figure 1J,K). These findings establish ARL3 as a luminal lineage‐enriched oncoprotein and support its potential as a biomarker for breast cancer progression.

### 2.2 ARL3 Deletion Suppresses Proliferation, Migration, and Enhances Endocrine Sensitivity in Hormone Receptor‐Positive Breast Cancer In Vitro

**Figure 2:**
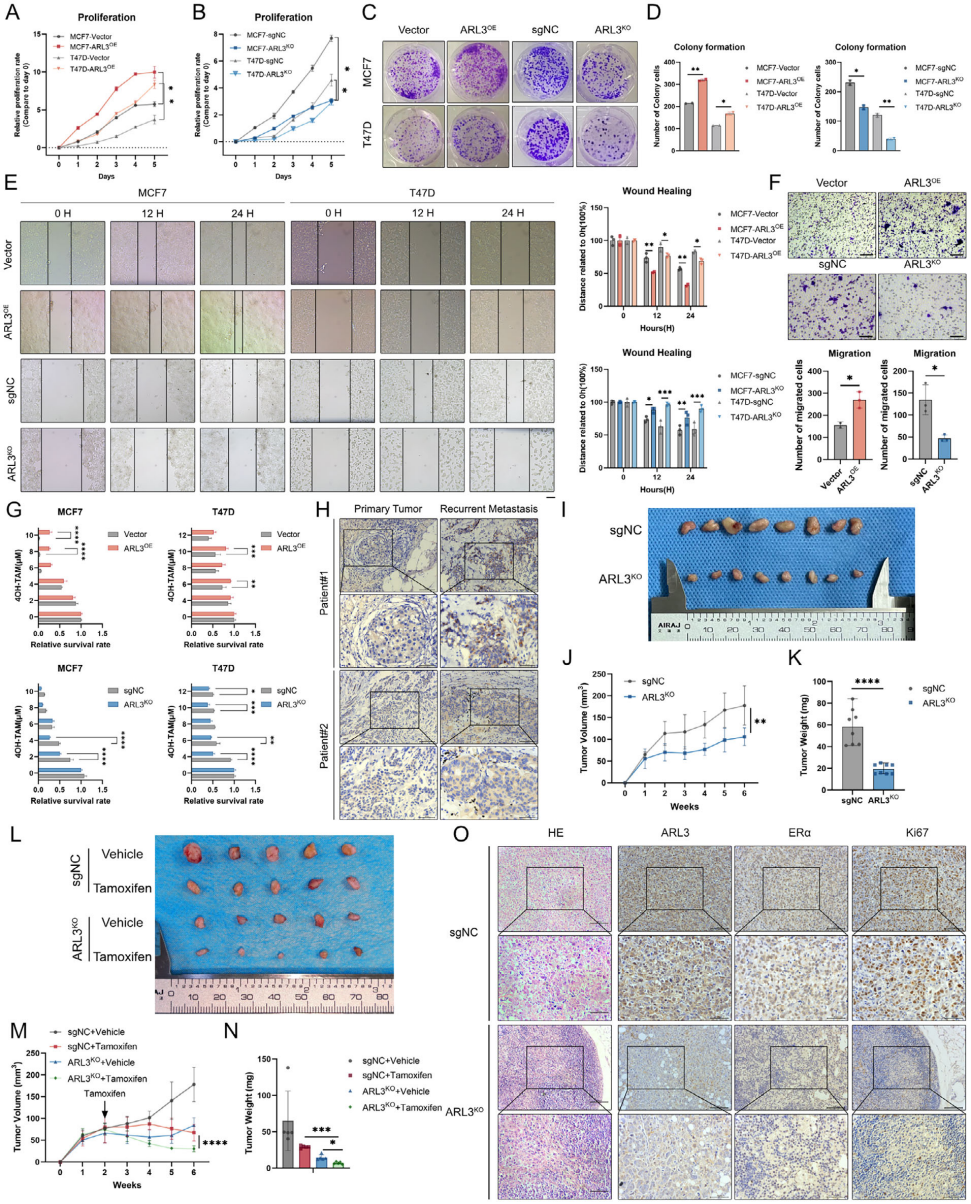
ARL3 knockout suppresses progression and enhances tamoxifen sensitivity in HR+ breast cancer in vitro and in vivo. A,B) Cell viability assessed by CCK‐8 assay in ARL3‐modified MCF7(A) and T47D cells (B). Data represent mean ± SD (*n* = 3 technical replicates). Significant differences were analyzed by paired two‐tailed Student's *t*‐test (^*^ *p* < 0.05). C,D) Colony formation assay demonstrating proliferative capacity of ARL3‐overexpressing and knockout cells. Data shown as mean ± SD (*n* = 3). One‐way ANOVA (^*^ *p* < 0.05, ^**^ *p* < 0.01). E,F) Impairment of migratory capacity by ARL3 knockout as evidenced by wound healing assays (E) and transwell migration (F). Data presented as mean ± SD (*n* = 3 independent experiments). Statistical analysis performed using two‐way ANOVA in E, paired two‐tailed Student's *t*‐test (^*^ *p* < 0.05, ^**^ *p* < 0.01, ^***^ *p* < 0.001). Scare bars, 250 µm (wound healing), 75 µm (transwell). G) CCK‐8 assay for evaluating tamoxifen resistance in transfected MCF7 and T47D cells following treatment with the indicated concentrations of tamoxifen for 48 h. Mean (*n* = 3) ± s.d. Two‐way ANOVA (^*^ *p* < 0.05, ^**^ *p* < 0.01, ^***^ *p* < 0.001, ^***^ *p* < 0.0001). H) Immunohistochemical analysis of ARL3 in primary tumors and relapsed metastatic tissues following endocrine therapy. Scare bars, 100 µm(upper), 50 µm(lower). I) Representative images displaying xenografts in nude mice of the sgNC and ARL3‐KO group. J,K) Growth curves and weights of xenograft tumors, Mean (*n* = 8) ± s.d. Two‐way ANOVA in J, Student's *t*‐test in K (^**^ *p* < 0.01, ^****^ *p* < 0.0001). L) Representative images displaying xenografts in nude mice of vehicle and tamoxifen treatment. M,N) Growth curves and weights of xenograft tumors subjected to the indicated treatments, Mean (*n* = 5) ± s.d. Two‐way ANOVA in M, one‐way ANOVA in N (^*^ *p* < 0.05, ^***^ *p* < 0.001). O) H&E staining and representative immunohistochemistry (IHC) images showing ARL3 (brown cytoplasm staining), ERα (brown nuclear staining), and Ki67‐positive cells (brown nuclear staining) in treated tumors. Scale bar, 100 µm (top), 50 µm(bottom).

Building on preliminary findings indicating ARL3's potential role in modulating the biological behavior of hormone receptor‐positive (HR+) breast cancer cells, we generated stable cell lines with constitutive ARL3 overexpression and CRISPR/Cas9‐mediated knockout. Rigorous validation via quantitative PCR (qPCR) and Western blot (WB) confirmed successful modulation of ARL3 expression, providing a robust foundation for subsequent functional analyses (Figure S2A–C, Supporting Information). Then we performed CCK8 proliferation assays and clonogenic survival tests, which demonstrated that ARL3 overexpression significantly promoted cell growth and colony formation, whereas ARL3 knockout attenuated these processes (**Figure** **2**A–D). Wound‐healing and transwell migration assays further showed that ARL3 downregulation impaired breast cancer cell motility, while ectopic ARL3 expression enhanced migratory capacity (Figure 2E,F). Notably, when assessing ARL3 function in triple‐negative breast cancer (TNBC) cell lines, overexpression of ARL3 had no discernible impact on cellular proliferation, indicating that its oncogenic effects are specific to the HR+ molecular subtype (Figure S2D–F, Supporting Information). Given the central role of endocrine therapy in HR+ breast cancer management, we evaluated the sensitivity of ARL3‐modified cells to tamoxifen, a first‐line endocrine agent. Consistent with bioinformatic analyses, ARL3‐knockout cells exhibited enhanced sensitivity to tamoxifen, whereas ARL3‐overexpressing cells displayed modestly reduced drug responsiveness (Figure 2G). To validate these in vitro observations, immunohistochemical staining of clinical samples from tamoxifen‐resistant recurrent/metastatic patients revealed significantly higher ARL3 expression in relapsed tumor tissues compared to matched primary lesions (Figure 2H). Collectively, these results establish ARL3 as a critical regulator of HR+ breast cancer progression, mediating cell proliferation, migration, and endocrine therapy sensitivity. The specificity of ARL3‐dependent effects to HR+ contexts highlights its potential as a subtype‐selective therapeutic target in this disease subset.

### 2.3 ARL3 Knockout Suppresses Proliferation and Increases the Tamoxifen Sensitivity of HR+ BC Cells In Vivo

To systematically investigate the functional significance of ARL3 in modulating proliferative capacity and endocrine therapeutic responsiveness in hormone receptor‐positive (HR+) breast malignancies, we established a syngeneic orthotopic transplantation model using HR+ breast cancer cells with stable ARL3 knockout (ARL3‐KO) or control (sgNC) expression. In vivo tumorigenicity assays demonstrated that ARL3‐KO cells exhibited profoundly attenuated tumor growth, with xenografts displaying significant reductions in both volumetric expansion (Figure 2I,J) and final tumor weight (Figure 2K) compared to controls. Strikingly, pharmacological intervention with tamoxifen revealed enhanced therapeutic responsiveness in ARL3‐deficient tumors, manifested in smaller lesion dimensions and lower tumor weights compared to tamoxifen‐treated controls (Figure 2L–N). Subsequent immunohistopathological analysis demonstrated a reduction of estrogen receptor‐α (ERα) nuclear staining and Ki67 proliferative indices in ARL3‐KO specimens (Figure 2O). These multimodal findings establish ARL3 as a critical regulator of HR+ breast cancer progression and endocrine resistance mechanisms in vivo.

### 2.4 ARL3 Drives HR+ Breast Cancer Progression via ERα‐Dependent Signaling Activation

**Figure 3:**
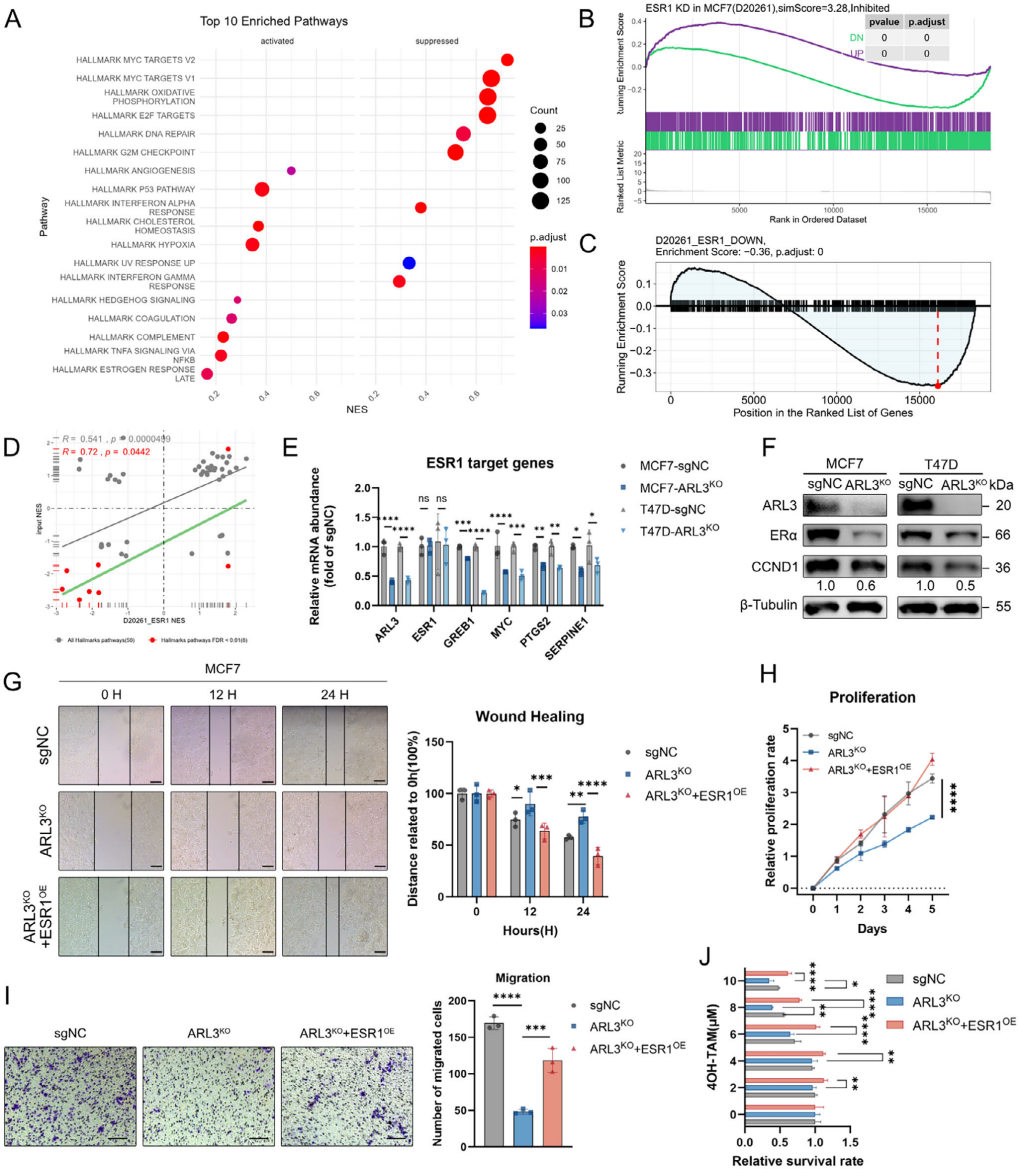
ARL3 promotes tamoxifen resistance by increasing ESR1 expression. A) KEGG analysis of RNA‐seq (sgNC vs ARL3‐KO). B–D) GSEA plots (B,C) and GPSA (D) correlation diagrams generated from both the ESR1‐KD dataset obtained through the GPSA online platform and our corresponding RNA‐seq analysis. E,F) Quantitative PCR analysis of mRNA expression and Western blot detection of protein levels in the estrogen receptor (ER) downstream signaling pathway following ARL3 knockout (ARL3‐KO). G‐J) Functional assays assessing wound healing (G), cellular proliferation (CCK‐8, H), transwell assay(I), and tamoxifen sensitivity (J) following ESR1 reconstitution in ARL3‐knockout (ARL3‐KO) MCF7 cells. Mean (*n* = 3) ± s.d. Two‐way ANOVA (^*^ *p* < 0.05, ^**^ *p* < 0.01, ^***^ *p* < 0.001, ^***^ *p* < 0.0001). Scare bars, 250 µm (wound healing), 75 µm (transwell).

For elucidating the mechanisms by which ARL3 affects the progression of HR‐positive breast cancer, we conducted RNA‐sequencing (RNA‐seq) on ARL3‐knockout (ARL3‐KO) and isogenic control MCF7 cells, followed by functional enrichment analysis. Transcriptomic profiling revealed that ARL3 deficiency significantly repressed gene sets associated with the MYC oncogenic network, E2F transcriptional targets, and G2/M cell cycle checkpoints—canonical downstream pathways of estrogen receptor 1 (ESR1, **Figure** **3**A; Figure S3A–D, Supporting Information). Given the central role of estrogen receptor α (ERα) signaling in HR+ tumorigenesis,^[^ ^28^ ^]^ we hypothesized that ARL3 modulates ERα‐dependent transcriptional programs. Exploring the functional interplay between ARL3 and ESR1, we leveraged the Genetic Perturbation Similarity Analysis (GPSA) platform (http://guotosky.vip:13838/GPSA/) to compare our ARL3‐KO transcriptome with publicly available ESR1‐knockdown datasets in MCF7 cells. Pearson correlation analysis demonstrated a significant positive similarity in gene expression signatures between ARL3 depletion and ESR1 loss (r = 0.72, *p* < 0.042), validated by overlapping enrichment of estrogen‐responsive pathways and differential expression of shared target genes (Figure 3B–D). Public dataset mining (TCGA, GSE2607) also showed elevated ARL3 levels in ERα‐positive versus ERα‐negative breast tumors (Figure S3E,F, Supporting Information), indicating ARL3 plays an important role in regulating ERα expression. Clinical correlation analysis revealed a significant positive association between ARL3 and ESR1 expression (Figure S3G, Supporting Information). To investigate the regulatory relationship between ARL3 and ERα, we first determined their upstream‐downstream relationship. We confirmed that ARL3 mRNA levels remained unchanged following ESR1 knockout (ESR1‐KO) in cells, indicating that ESR1 acts downstream of ARL3 (Figure S3H, Supporting Information). Quantitative real‐time PCR (qRT‐PCR) confirmed reduced mRNA levels of ERα downstream effectors—GREB1, MYC, PTGS2, and SERPINE1—in ARL3‐KO cells, whereas ESR1 transcript levels remained unaltered (Figure 3E). Western blot analysis further revealed decreased protein abundance of ERα and its downstream cell‐cycle regulator CCND1, without changes in ESR1 transcription, indicating post‐translational regulation of ERα signaling by ARL3 (Figure 3F). Meanwhile, we demonstrated through immunofluorescence and Western Blot (WB) that the nuclear localization of ERα decreased after ARL3 knockout (ARL3‐KO), indicating that ARL3 can affect the nucleocytoplasmic migration of ERα (Figure S3I,K, Supporting Information). To establish causality, rescue experiments were performed by ectopically overexpressing ERα in ARL3‐KO cells. Forced ERα expression fully restored proliferation (CCK8 assay and colony formation), migratory capacity (wound‐healing assay), and attenuated tamoxifen sensitivity, phenocopying the effects of wild‐type cells (Figure 3G–J). Taken together, our data demonstrate that ARL3 exerts its biological effects in HR+ breast cancer through post‐transcriptional regulatory mechanisms of ERα.

### 2.5 ARL3 Enhance ERα Protein Stability through by Regulating K48‐ and K63‐Linked Polyubiquitination at the K252 Site

**Figure 4:**
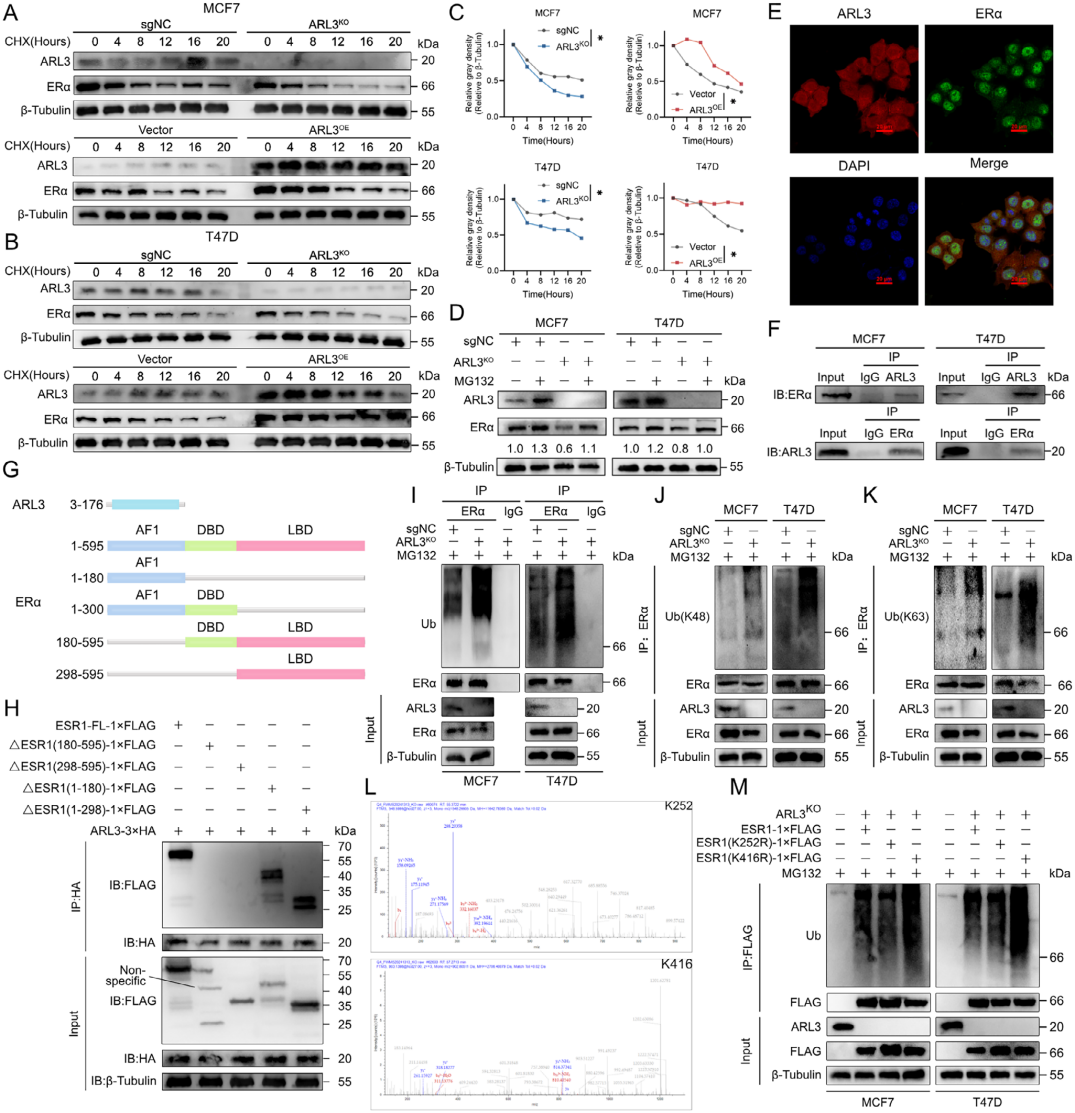
Knockout of ARL3 Promotes Degradation of ERα via Enhancing Polyubiquitination at K252 residue. A–C) Cycloheximide (CHX, 100 µg mL^−1^) chase assays in ARL3‐overexpressing and knockout cells. Quantified ERα decay curves with calculated half‐lives (C) (Mean (*n* = 3) ± s.d. Student's *t*‐test (^*^ *p* < 0.05). β‐Tubulin serves as loading control. D) Immunoblots showing MG132 (10 µm, 6 h) rescues ERα levels in ARL3 knockout cells. β‐Tubulin serves as a loading control. E) Representative confocal images showing ARL3 (red) and ERα (green) co‐localization in MCF7 cells. Nuclei were counterstained with DAPI (blue). Scale bar, 20 µm. F) Co‐IP assays of ARL3 and ERα in MCF7 and T47D cells. Input was shown as an expression control. G,H) Schematic outlines of ARL3 and ESR1 structural features (G). The indicated constructs were transfected into HEK293T cells. After 24 h, cells were subjected to immunoprecipitation (IP) using anti‐FLAG or anti‐HA antibodies (H). I–K) MCF7 and T47D cells were co‐transfected with the designated plasmids and treated with MG132 prior to cell lysis. Immunoprecipitation (IP) was performed on cell lysates using an anti‐ERα antibody, followed by Western blotting (WB) with antibodies targeting ubiquitin (Ub) (I), Lys48 (K48)‐linked ubiquitin (J), Lys63 (K63)‐linked ubiquitin (K), and ERα. L) Ubiquitination mapping in ARL3‐deficient MCF7 cells, identifying characteristic ubiquitin signatures at K252 and K416 (arrows). M) Denaturing immunoprecipitation analysis of wild‐type (WT) ESR1 and lysine‐to‐arginine mutants (K252R, K416R) in ARL3 knockout (KO) cells.

Given that previously discovered that ARL3 potentially impact ERα protein levels, we confirmed that ARL3 does not affect protein synthesis (Figure S3J, Supporting Information). We hypothesized that ARL3 might affect the degradation of ERα protein. Next, we assessed ERα protein stability was evaluated via cycloheximide (CHX) chase experiments in control, ARL3‐knockout (ARL3‐KO), and ARL3‐overexpressing (ARL3‐OE) cells. ERα half‐life was prolonged in ARL3‐OE cells and shortened in ARL3‐KO cells (**Figure** **4**A–C). We also found rescue with the proteasome inhibitor MG132, but not the lysosome inhibitor chloroquine (CQ), restored ERα levels in ARL3‐KO cells (Figure 4D; Figure S3L, Supporting Information), indicating proteasome‐dependent regulation by ARL3. Then, we employed immunofluorescence microscopy and observed colocalization of ARL3 and ERα in both nuclear and cytoplasmic compartments (Figure 4E), with co‐immunoprecipitation (Co‐IP) assays confirming direct physical interaction (Figure 4F).

Considering the observation that ARL3 regulates ERα protein degradation, we next aimed to investigate whether this effect involves an interaction between the two proteins. To identify the specific domains mediating their association, we conducted truncation mutant interaction assays. First, we mapped the interaction interface, truncated constructs of ARL3 (GTPase domain) and ERα [activation function 1 (AF1), DNA‐binding (DBD), ligand‐binding (LBD) domains] were generated (Figure 4G). Co‐IP experiments identified the ERα ligand‐binding domain (LBD) as the critical interaction motif for ARL3 (Figure 4H). It potentially interferes with the binding of estradiol (E2) to ERα. This interference could disrupt the normal process of E2‐induced nuclear translocation of ERα and ultimately lead to metabolic reprogramming.^[^ ^23^, ^29^ ^]^

We next sought to determine how this interaction influences ERα protein stability. We hypothesized that ARL3 might regulate ERα turnover by affecting its ubiquitination status. To test this, we performed ubiquitination‐specific IP assays and revealed increased total ubiquitination of ERα in ARL3‐KO cells (Figure 4I). Previous studies have established that K48‐ and K63‐linked ubiquitin chains are primarily associated with proteasomal degradation and non‐degradative signaling processes, respectively, while the biological functions of K6‐ and K11‐linked ubiquitination remain less characterized in comparison.^[^ ^30^ ^]^ Using K48‐ and K63‐specific antibodies, we found that ARL3 depletion enhanced K48‐ and K63‐linked ubiquitination of ERα (Figure 4J,K), driving the K48 chain leads degradation to reduce abnormal ERα, while the K63 chain attempts to maintain its signaling function. The majority of ubiquitination events occur at lysine residues, which serve as the primary acceptor sites for ubiquitin conjugation.^[^ ^31^ ^]^ We first performed bioinformatic analysis via the GPS‐Uber platform predicted conserved ubiquitination sites at ESR1 lysine residues K252 and K416 across vertebrates (Figure S4A,B, Supporting Information), and ubiquitin mapping in ARL3‐deficient MCF7 cells validated modification at these positions (Figure 4L). Functional validation using lysine‐to‐arginine (K/R) mutants revealed that the K252R substitution, but not K416R, attenuated ERα ubiquitination in ARL3‐KO cells (Figure 4M), indicating preferential regulation of K252‐linked polyubiquitination by ARL3. This differential modification pattern suggests ARL3 preferentially regulates the polyubiquitination status at K252 residue rather than K416. Collectively, these findings reveal that ARL3 interacts with the ligand‐binding domain of ERα to suppress K48‐ and K63‐linked polyubiquitination—predominantly at the K252 residue—thereby regulating ERα proteasomal degradation and functionally influencing its nuclear translocation and downstream metabolic reprogramming.

### 2.6 ARL3 Stabilizes ERα by Recruiting USP10 to Attenuate K48/K63‐Linked Polyubiquitination

**Figure 5:**
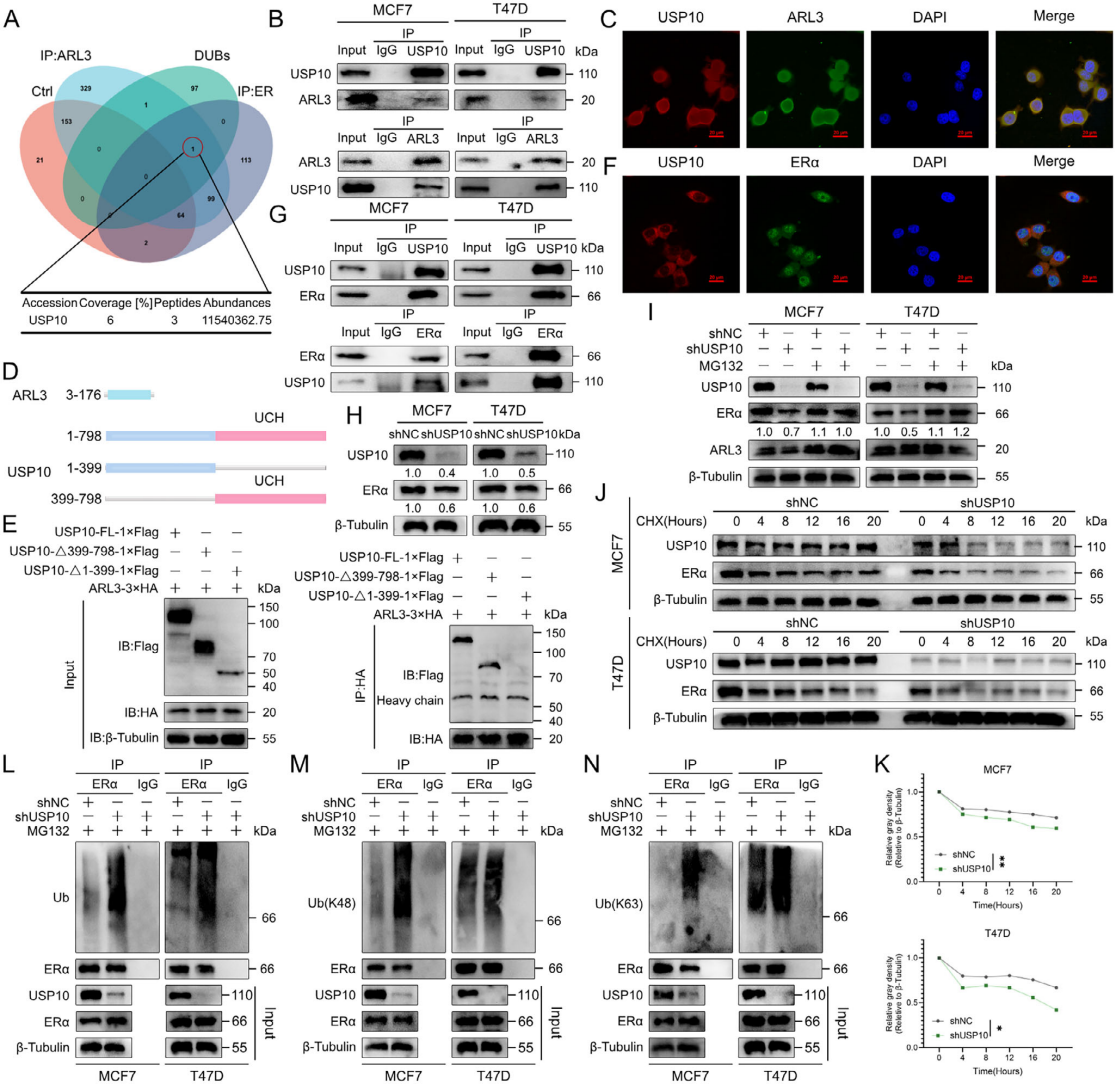
ARL3 Induces USP10 to Deubiquitylate and Stabilize ERα. A) Venn diagram of overlapping candidates from Ctrl (red), ARL3 interactome (blue), DUBs (green), and cytoplasmic ERα proteome (dark blue). B) Co‐IP assays of USP10 and ARL3 in MCF7 and T47D cells. Input (10% lysate) was shown as an expression control. C) Confocal images showing cytoplasmic co‐localization (yellow puncta) of endogenous USP10 (red) and ARL3 (green) in MCF7 cells. Scale bar, 20 µm. D,E) Schematic outlines of ARL3 and USP10 structural features (D). The indicated constructs were transfected into HEK293T cells. After 24 h, cells were subjected to immunoprecipitation (IP) using anti‐FLAG or anti‐HA antibodies (E). F) Confocal images showing cytoplasmic co‐localization (yellow puncta) of endogenous USP10 (red) and ERα (green) in MCF7 cells. Scale bar, 20 µm. G) Co‐IP assays of USP10 and ERα in MCF7 and T47D cells. Input (10% lysate) was shown as an expression control. H) Immunoblots showing reduction of ERα protein levels in USP10‐KD MCF7 and T47D cells versus shNC. β‐Tubulin serves as a loading control. I) Immunoblots showing MG132 (10 µm, 6 h) rescues ERα levels in USP10‐KD cells. β‐Tubulin serves as a loading control. J,K) Cycloheximide (CHX, 100 µg mL^−1^) chase assays in USP10‐KD cells. Quantified ERα decay curves with calculated half‐lives (K) (Mean (*n* = 3) ± s.d. Student's *t*‐test (^*^ *p* < 0.05, ^**^ *p* < 0.01). β‐Tubulin serves as loading control. L–N) MCF7 and T47D cells were co‐transfected with the designated plasmids and treated with MG132 prior to cell lysis. Immunoprecipitation (IP) was performed on cell lysates using an anti‐ERα antibody, followed by Western blotting (WB) with antibodies targeting ubiquitin (Ub) (L), Lys48 (K48)‐linked ubiquitin (M), Lys63 (K63)‐linked ubiquitin (N), and ERα.

Our findings establish ARL3 as a critical post‐translational regulator of ERα, suppressing its polyubiquitination to maintain protein stability. To unravel the molecular mechanisms underlying this process, we hypothesized that ARL3 might engage deubiquitinating enzymes (DUBs) to counteract ERα ubiquitination—a well‐documented regulatory axis for oncogenic transcription factors.^[^ ^16^, ^32^, ^33^ ^]^ Integrative analysis of ARL3 interactomes and cytoplasmic ERα proteomic datasets identified ubiquitin‐specific protease 10 (USP10)—a DUB with established roles in tumorigenesis but uncharacterized in ERα biology—as the top candidate (**Figure** **5**A; Figure S4C and Tables S1 and S2, Supporting Information). Co‐immunoprecipitation assays confirmed a direct interaction between USP10 and ARL3 (Figure 5B). Furthermore, their co‐localization in the cytoplasm was visualized by immunofluorescence confocal microscopy (Figure 5C). To map the specific domain of USP10 responsible for this interaction, we generated a series of USP10 truncation constructs (Figure 5D). Subsequent Co‐IP experiments revealed that ARL3 specifically interacts with the 1–399 amino acids in the N‐terminal fragment of USP10 (Figure 5E).

Then we demonstrated that ARL3 knockout reduced USP10 protein levels without mRNA change (Figure S4D,E, Supporting Information), indicating that the regulation occurs at the post‐transcriptional level. Moreover, immunofluorescence analysis demonstrated substantial cytoplasmic co‐localization between USP10 and ERα, mirroring the subcellular distribution of ARL3 (Figure 5F), suggesting a cytoplasmic complex formation. This spatial concordance suggests a cytoplasmic quality control module wherein ARL3 scaffolds USP10 to counteract ERα ubiquitination—a hypothesis corroborated by reciprocal co‐immunoprecipitation assays demonstrating direct ARL3‐USP10‐ERα ternary complex formation (Figure 5G). USP10 knockdown markedly decreased ERα protein levels without altering its mRNA expression (Figure 5H; Figure S4F, Supporting Information). Functional stability assays showed that proteasome inhibition with MG132 rescued ERα levels in USP10‐KD cells (Figure 5I), and cycloheximide (CHX) chase experiments demonstrated a marked reduction in ERα half‐life upon USP10 depletion—phenocopying the destabilization observed in ARL3‐KO cells (Figure 5J,K). Consistent with ARL3's role in suppressing ERα polyubiquitination, USP10‐KD also increased K48‐ and K63‐linked ubiquitin chain accumulation on ERα (Figure 5L–N), reinforcing the functional overlap between ARL3 and USP10 in regulating ERα ubiquitination status. These results define a mechanistic pathway wherein ARL3 recruits USP10 to deubiquitinate ERα, specifically reducing K48‐ and K63‐linked polyubiquitination to antagonize proteasomal degradation.

### 2.7 ARL3 Deficiency Impairs Mitochondrial Function via ERα‐Mediated Mitophagy Regulation

**Figure 6:**
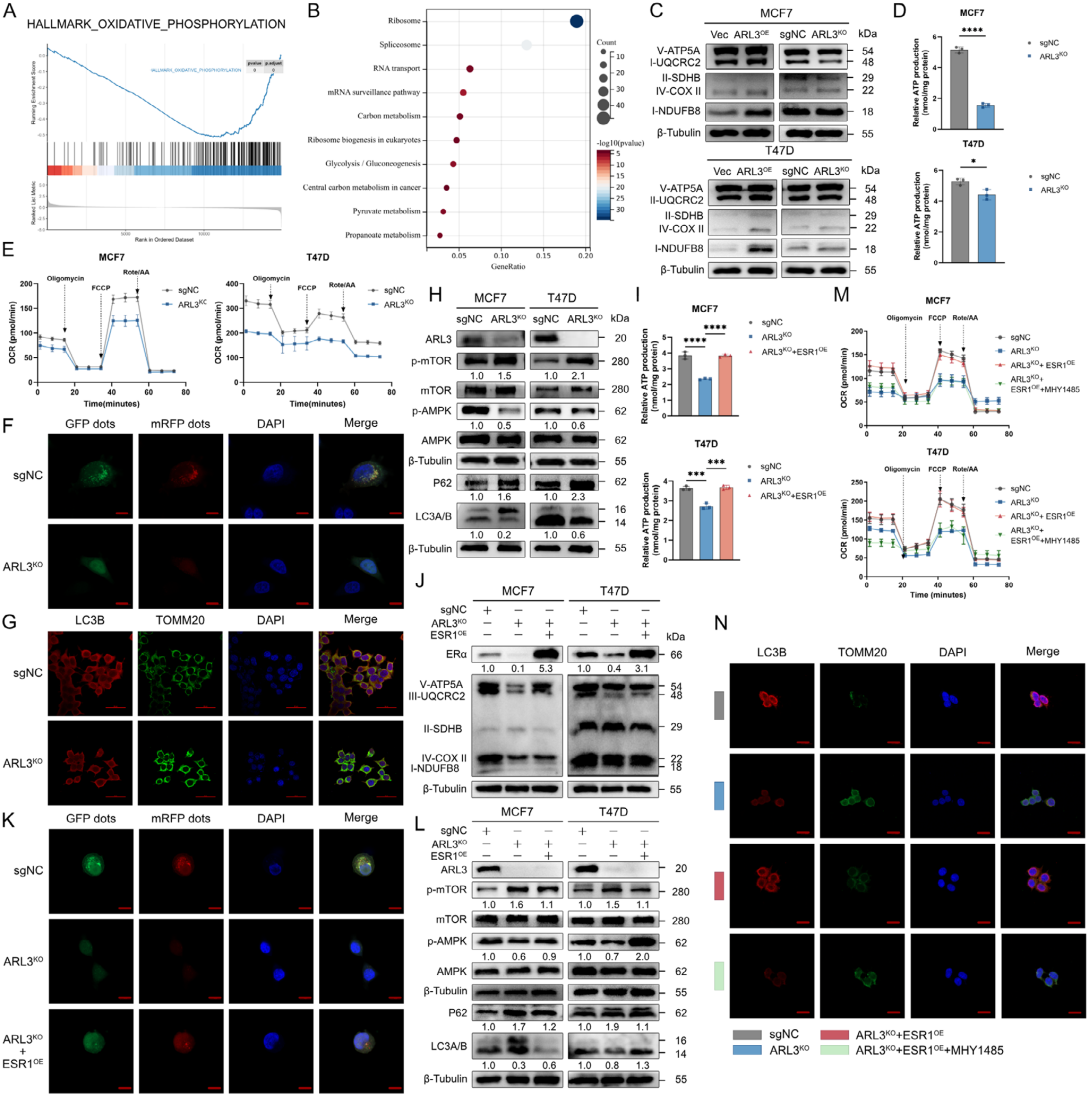
ARL3 promotes mitophagy through ERα upregulation. A) Gene Set Enrichment Analysis (GSEA) of RNA‐seq data (sgNC VS ARL3‐KO). B) GO term enrichment analysis of ARL3‐interacting proteins identified by immunoprecipitation‐mass spectrometry (IP/MS). C) Immunoblots of OXPHOS complex subunits (CI‐NDUFB8, CII‐SDHB, CIII‐UQCRC2, CIV‐MTCO1, CV‐ATP5A) in MCF7 and T47D cells transfected with ARL3 overexpression (OE) or knockout (KO) plasmids. β‐Tubulin serves as a loading control. D,E) Mitochondrial functional profiling in sgNC, ARL3‐KO cells, ATP production (D), and seahorse analysis (E). F,G) Autophagic flux analyzed by mRFP‐GFP‐LC3 reporter via confocal microscopy. Scare bars, 20 µm (F). Representative confocal images showing LC3B (red) and TOMM20 (green) co‐localization in sgNC, ARL3‐KO cells. Nuclei were counterstained with DAPI (blue). Scale bar, 50 µm (G). H) Representative immunoblots of p‐mTOR (Ser2448)/total mTOR, p‐AMPKα (Thr172)/total AMPKα, p62, LC3B‐I/II, and β‐tubulin (loading control) in sgNC, ARL3‐KO cells. β‐Tubulin serves as loading control. I) Cellular ATP levels in sgNC, ARL3‐KO, ARL3‐KO+ESR1‐OE MCF7, and T47D cells. Mean (*n* = 3) ± s.d. Two‐way ANOVA (^***^ *p* < 0.001, ^****^ *p* < 0.0001). J) Immunoblots of OXPHOS complex subunits (CI‐NDUFB8, CII‐SDHB, CIII‐UQCRC2, CIV‐MTCO1, CV‐ATP5A) in sgNC, ARL3‐KO, ARL3‐KO+ESR1‐OE MCF7 and T47D cells. β‐Tubulin serves as a loading control. K) Autophagic flux analysis using mRFP‐GFP‐LC3 reporter. Yellow puncta (mRFP+GFP+) represent autophagosomes, red puncta (mRFP+GFP‐) indicate autolysosomes. Scale bar, 20 µm. L) Immunoblots of p‐mTOR, total mTOR, p‐AMPKα, total AMPKα, p62, and LC3B‐I/II in sgNC, ARL3‐KO, ARL3‐KO+ESR1‐OE cells. β‐Tubulin serves as a loading control. M) Seahorse analysis of sgNC, ARL3‐KO, ARL3‐KO+ESR1‐OE, and ARL3‐KO+ESR1‐OE +MHY1485 in MCF7 and T47D cells. N) Representative confocal images showing LC3B (red) and TOMM20 (green) co‐localization in sgNC, ARL3‐KO, ARL3‐KO+ESR1‐OE, and ARL3‐KO+ESR1‐OE +MHY1485 cells. Nuclei were counterstained with DAPI (blue). Scale bar, 20 µm.

Tumor cells adapt to microenvironmental stress and sustain proliferation through metabolic reprogramming, a process closely linked to tumor progression and chemoresistance.^[^ ^34^, ^35^ ^]^ Previous studies found clinically relevant LBD mutations (detected in ≈20% of metastatic ER+ cases) confer resistance to both selective estrogen receptor modulators (SERMs, e.g., tamoxifen) and degraders (SERDs, e.g., fulvestrant) through impaired ligand binding,^[^ ^29^, ^36^ ^]^ and they also contribute to tamoxifen resistance through metabolism dysfunction of breast cancer.^[^ ^23^ ^]^ Since we unexpectedly discovered that ARL3 and ERα has an interaction in the LBD domain (Figure 4H). Integrating our subsequent results, the combined transcriptomic and proteomic profiling disclosed that ARL3 deficiency induces metabolic reprogramming in HR+ breast cancer. Specifically, RNA‐seq/GSEA analysis revealed a significant suppression of the oxidative phosphorylation (OXPHOS) pathways (**Figure** **6**A), and mass spectrometry identified ARL3 interactomes that were enriched in central carbon metabolism (Figure 6B). Western blot validation demonstrated increased levels of mitochondrial respiratory chain complexes (Complexes I–IV) in ARL3‐overexpressing cells, whereas ARL3‐KO cells exhibited reduced expression of these proteins (Figure 6C), confirming direct effects of ARL3 on OXPHOS machinery. Moreover, measurements of mitochondrial ATP production and Seahorse assays demonstrated that ATP levels were significantly reduced in ARL3‐KO cells while induced in ARL3‐OE cells (Figure 6D,E; Figure S4G–J, Supporting Information).

Considering the previously reported mitochondrial localization of ARL3 and its connection with autophagic organelles,^[^ ^37^ ^]^ we evaluated autophagic flux using LC3 plasmid transfection and confocal microscopy. ARL3‐KO cells displayed reduced yellow fluorescence (indicating autophagosome‐lysosome fusion), signifying impaired autophagic maturation compared to controls (Figure 6F). Given the predominant mitochondrial localization of ARL3, we further performed immunofluorescence co‐staining of LC3B and the mitochondrial marker TOMM20 (Figure 6G). The results demonstrated decreased colocalization between TOMM20 and LC3B in ARL3‐KO cells, implying a potential concurrent suppression of mitophagy. Western blot analysis of autophagy‐related proteins confirmed that ARL3‐KO cells exhibited increased phosphorylated mTOR (p‐mTOR), decreased phosphorylated AMPK (p‐AMPK), and a reduced LC3B‐II/LC3B‐I ratio, collectively indicating dysregulated mTOR/AMPK signaling and attenuated autophagic flux (Figure 6H). Subsequent transmission electron microscopy (TEM) analysis further visualized abnormal mitochondrial morphology and accumulation of damaged organelles in ARL3‐KO cells, consistent with defective mitophagic clearance (Figure S4K, Supporting Information). This mitochondrial quality control failure likely results in reduced ATP production, thereby creating a bioenergetic crisis that compromises tumor cell viability and progression.

To determine whether ERα mediates ARL3‐dependent mitochondrial regulation, we performed ESR1 rescue experiments in ARL3‐depleted cells. Ectopic ERα expression restored OXPHOS complex protein levels and rescued mitochondrial ATP production deficits induced by ARL3 knockout (Figure 6I,J). Confocal microscopy analysis demonstrated that ERα reconstitution reversed the autophagy suppression observed in ARL3‐KO cells, as evidenced by normalized LC3B puncta formation and restored colocalization with lysosomal markers (Figure 6K). Biochemical analyses showed that ERα rescue rebalanced the mTOR/AMPK axis, reducing p‐mTOR and increasing p‐AMPK levels to control‐like states (Figure 6L).

Based on the results, we hypothesize that ARL3 modulates mitochondrial function by regulating the AMPK/mTOR signaling pathway through ERα. To validate this hypothesis, we performed a pathway rescue experiment in ARL3‐knockout cells with reconstituted ERα using the mTOR agonist MHY1485, where reconstitution of ERα had suppressed mTOR signaling. Following MHY1485 treatment, Seahorse metabolic flux analysis (Figure 6M; Figure S4L,M, Supporting Information) and immunofluorescence staining for TOMM20 and LC3B demonstrated that mTOR pathway activation restored mitochondrial respiration, suppressed LC3B signal, and enhanced TOMM20‐positive puncta formation, suggesting attenuated autophagy and increased mitochondrial mass (Figure 6N), thereby inhibiting the ERα reconstitution‐induced mitophagy. Concurrently, the expression levels of OXPHOS complex proteins were restored (Figure S4N, Supporting Information). These findings compellingly demonstrate that the ERα‐modulated mTOR pathway serves as the key mediator through which ARL3 regulates mitochondrial bioenergetics and mitophagic flux.

### 2.8 Pharmacological Inhibition of ARL3 Destabilizes ERα to Suppress Tumor Growth and Sensitize Endocrine Therapy

**Figure 7:**
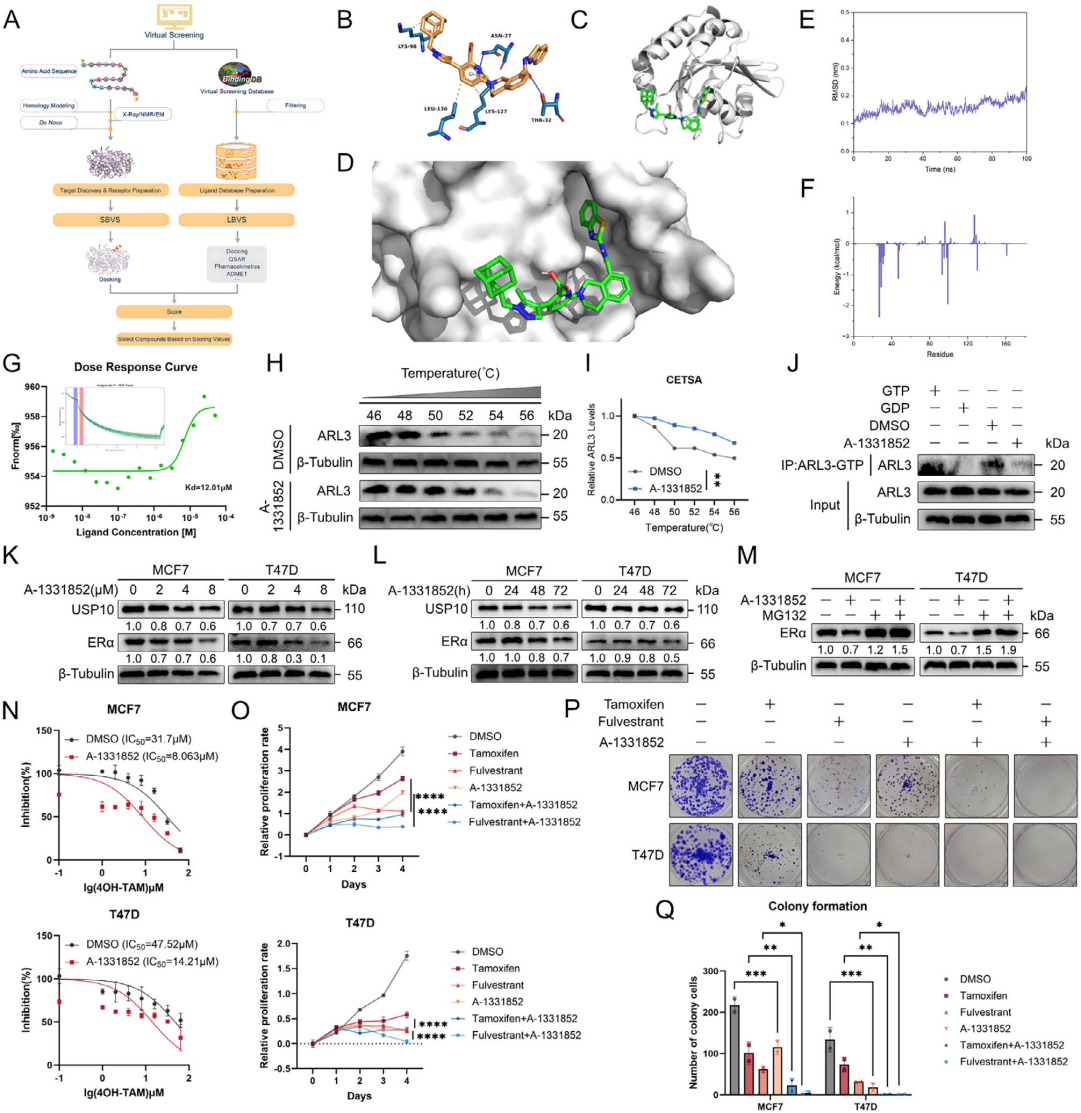
Pharmacological targeting of ARL3 disrupts the ARL3/USP10/ERα axis and synergizes with endocrine therapy in HR+ breast cancer. A–D) Structure‐based virtual screening of 15 000 FDA‐approved compounds in the L‐CO‐020 library, identifying top ARL3 GTPase domain‐binding candidates (A, screening workflow; B, Chemical structures of prioritized compounds; C, Binding conformation of protein ARL3 to A‐1331852 Small molecule (cartoon); D, Enlarged conformation of ARL3 binding A‐1331852 small molecule (surface). E) RMSD diagram of molecular dynamics of the complex. F) Binding free energy of the molecular dynamics of the complex. G) Binding affinity between A‐1331852 and ARL3. H,I) CTESA assay of ARL3 stability in DMSO and A‐1331852‐treated cells. (H) Western blot analysis of ARL3 protein levels in cells treated with DMSO or A‐1331852 at various temperatures. (I) Quantified decay curves of ARL3 protein across temperatures, derived from panel H (Student's *t*‐test, ^**^ *p* < 0.01). β‐Tubulin serves as a loading control. J) Immunoprecipitation (IP) was performed on cell lysates using an anti‐active ARL3 antibody, followed by Western blotting (WB) with antibodies targeting ARL3 treated with GTP (positive control), GDP (negative control), DMSO, and A‐1331852. K,L) Immunoblot analysis of ARL3, USP10, and ERα protein stability in HR+ breast cancer cells treated with A‐1331852, showing dose‐ (K) and time‐dependent (L) degradation (representative blots, quantitation normalized to β‐Tubulin). M) Immunoblot analysis of ERα protein in MCF7 and T47D cells treated with A‐1331852 and MG132. β‐Tubulin serves as a loading control. N) IC_5_₀ reduction of A‐1331852 + tamoxifen chemo‐sensitization in MCF7 and T47D cells. O) Cell viability assessed by CCK‐8 assay in A‐1331852, tamoxifen and fulvestrant treated MCF7 and T47D cells. Data represent mean ± SD (*n* = 3). Significant differences were analyzed by paired two‐tailed Student's *t*‐test (^****^ *p* < 0.0001). P,Q) Colony formation assay demonstrating proliferative capacity of tamoxifen and fulvestrant‐treated MCF7 and T47D cells. Data shown as mean ± SD (*n* = 3). One‐way ANOVA (^*^ *p* < 0.05, ^**^ *p* < 0.01, ^***^ *p* < 0.001).

Capitalizing on ARL3's established roles in driving oncogenesis, metastasis, and endocrine resistance in hormone receptor‐positive (HR+) breast cancer, we pursued pharmacological targeting of this pathway. Structure‐based virtual screening of 15 000 FDA‐approved compounds within the L‐CO‐020 library identified 20 candidates exhibiting high binding affinity for ARL3 (**Figure** **7**A–D; Tables S4–S6, Supporting Information). Following stringent filtering to eliminate solvent artifacts and fluorescent interferents, three lead compounds underwent functional validation for modulating the ARL3/USP10/ERα axis (Figure S5A, Supporting Information). Molecular dynamics (MD) simulations of the ARL3/A‐1331852 complex revealed biphasic behavior, initial structural fluctuations (0–20 ns) followed by stable equilibration (20–100 ns), characterized by minimal radius of gyration variation (<5%), persistent hydrogen bonding (8–9 bonds), and stable solvent‐accessible surface area (SASA) (Figure 7E, Figure S5B–E, Supporting Information). MM/GBSA calculations yielded a strong binding free energy of −33.29 kcal mol^−1^, corroborating the compound's high affinity for ARL3 (Figure 7F; Figure S5F–G, Supporting Information). MicroScale Thermophoresis (MST) confirmed direct binding between A‐1331852 and ARL3, with a dose‐response curve demonstrating concentration‐dependent binding and a dissociation constant (Kd) of 12.01 µm (Figure 7G). Subsequent Cellular Thermal Shift Assay (CETSA) under physiological conditions revealed enhanced thermal stability of ARL3 in A‐1331852‐treated cells compared to DMSO controls, indicating target engagement in situ (Figure 7H,I).

We further demonstrated that A‐1331852 inhibits ARL3 GTPase activity using an active‐state pulldown assay and immunofluorescence (Figure 7J; Figure S5H, Supporting Information). Immunoblot analysis showed this top‐ranked inhibitor induced dose‐ and time‐dependent depletion of USP10 and ERα proteins, consistent with predicted axis destabilization (Figure 7K,L), co‐treatment with MG132 abolished ERα downregulation, indicating proteasomal degradation mediates this effect (Figure 7M).

To establish ARL3 as the requisite target of A‐1331852 in HR+ breast cancer, we found that A‐1331852 exhibited no cytotoxic effects on MCF10A cells (Figure S5I, Supporting Information). We performed drug treatment in ARL3‐knockout (KO) cells. ARL3 ablation attenuated drug efficacy, confirming target dependency (Figure S5J, Supporting Information). Furthermore, genetic knockout of BCL‐2 and BCL‐XL demonstrated potent antiproliferative effects of A‐1331852 via CCK‐8 and colony formation assays, even upon complete loss of BCL‐XL/2 function (Figure S5K–P, Supporting Information). This confirms that A‐1331852's activity in HR+ breast cancer cells is not exclusively dependent on canonical BCL‐2 family inhibition and establishes its function as an ARL3 inhibitor.

Functional assays in HR+ cell lines demonstrated that A‐1331852 synergized with tamoxifen to enhance growth inhibition, while combination with fulvestrant led to pronounced suppression of neoplastic proliferation compared to monotherapies (Figure 7N–Q), highlighting its chemo‐sensitizing potential. Overall, these results suggest that aiming at ARL3 might selectively eliminate cells with elevated levels of both ARL3 and ERα expression.

**Figure 8:**
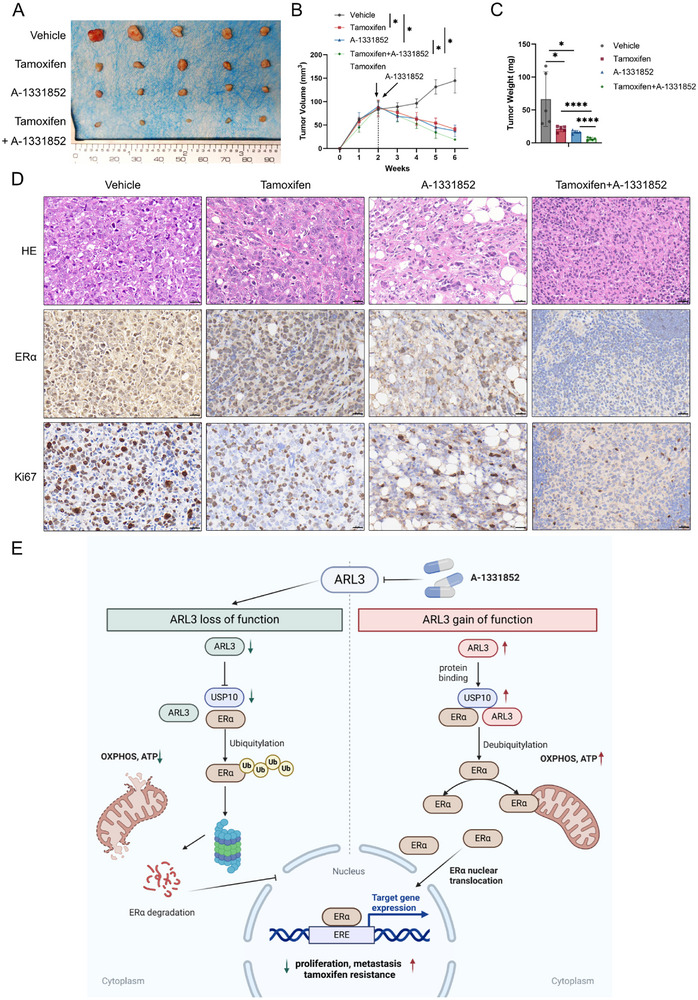
Combined ARL3 Inhibition and tamoxifen treatment suppresses HR+ breast cancer progression in vivo. A–C) In vivo therapeutic efficacy in orthotopic HR+ breast cancer models, (A) Representative xenograft images and (B) Tumor growth curves showing A‐1331852 monotherapy (25 mg kg^−1^) and combination‐driven regression;(C) tumor weight quantification post‐treatment. Data expressed as mean ± SD (*n* = 5 mice/group, Two‐way ANOVA in B, one‐way ANOVA in C, ^*^ *p* < 0.05, ^***^ *p* < 0.001). D) H&E staining and representative immunohistochemistry (IHC) images showing reduced ARL3 (brown cytoplasm staining), ERα (brown nuclear staining), and Ki67‐positive cells (brown nuclear staining) in treated tumors. Scale bar, 20 µm. E) Graphic abstract of this article.

To establish translational relevance, we evaluated A‐1331852's therapeutic efficacy in orthotopic HR+ breast cancer models. Pharmacological intervention (25 mg kg^−1^, literature‐based drug concentration) significantly attenuated tumor progression (**Figure** **8**A–C) through suppression of ARL3 and its downstream reduction of ERα and proliferation marker Ki67 expression level (Figure 8D), while combination therapy induced synergistic tumor regression when paired with endocrine agents. Toxicological assessment confirmed safety, as major organs exhibited no histopathological abnormalities (Figure S6A, Supporting Information). These multimodal findings position pharmacological ARL3 inhibition as a viable therapeutic strategy for dual‐pathway intervention‐simultaneously suppressing ESR1‐driven oncogenesis and enhancing endocrine therapy responsiveness in HR+ breast malignancies.

## 3 Discussion

Our study delineates a critical role for ARL3 in driving hormone receptor‐positive (HR+) breast cancer progression and endocrine resistance. Specifically, it uncovers a multifaceted mechanism whereby ARL3 stabilizes estrogen receptor α (ERα) through USP10‐mediated deubiquitination while orchestrating metabolic reprogramming via ERα‐dependent mitophagy.

In HR+ breast cancer, ERα remains the cornerstone of endocrine therapy, with its post‐translational regulation emerging as a key determinant of treatment response.^[^ ^38^, ^39^ ^]^ The ubiquitin‐proteasome system (UPS) finely tunes ERα abundance, E3 ligases such as RNF181 promote K63‐linked polyubiquitination to stabilize ERα,^[^ ^11^ ^]^  while deubiquitinating enzymes (DUBs) like USP36 and USP14 counteract proteasomal degradation by removing K48‐linked ubiquitin chains.^[^ ^19^, ^40^ ^]^  Our study identifies ARL3 as a previously unrecognized regulator of this equilibrium, functioning as a scaffold to recruit USP10 and suppress both K48‐ and K63‐linked polyubiquitination at the ERα K252 residue. This mechanism not only stabilizes ERα protein levels but also amplifies its downstream transcriptional programs, including MYC, E2F, and cell cycle checkpoints, which are central to tumor proliferation.^[^ ^6^, ^41^ ^]^ By binding to the ERα ligand‐binding domain (LBD)—a region implicated in mitochondrial localization^[^ ^29^, ^36^ ^]^—ARL3 integrates ERα signaling with subcellular compartmentalization, creating a nexus for oncogenic and metabolic regulation.

Tumor cells rely on metabolic adaptability to sustain growth under stress, a process often linked to therapy resistance.^[^ ^35^, ^42^ ^]^ Our discovery that ARL3 deficiency impairs oxidative phosphorylation (OXPHOS) and mitophagy through ERα‐dependent mechanisms reveals a novel axis connecting transcriptional and bioenergetic regulation. By modulating the AMPK/mTOR signaling cascade, ARL3 promotes mitophagic clearance of damaged mitochondria, ensuring mitochondrial quality control and ATP production critical for tumor cell survival.^[^ ^43^ ^]^ This dual role—stabilizing ERα to drive proliferative signaling while enhancing mitochondrial fitness—positions ARL3 as a master regulator of cellular homeostasis in HR+ breast cancer. The rescue of OXPHOS deficits via ERα overexpression in ARL3‐knockout cells underscores the interdependency of these pathways, suggesting that ARL3‐mediated metabolic reprogramming is both ERα‐dependent and essential for tumor progression.

Deubiquitinating enzymes like USP10 have emerged as key players in oncogenesis, often acting as tumor promoters by stabilizing oncoproteins.^[^ ^32^, ^44^ ^]^ In HR+ breast cancer, our identification of a ternary ARL3–USP10–ERα complex—mediated by the direct interaction between ARL3 and the USP10 (1–399AA) region—reveals a novel mechanism for ERα stabilization, distinct from those involving previously described deubiquitinases such as USP36.^[^ ^51^ ^]^ Importantly, this axis is selectively active in HR+ cells, as ARL3 overexpression fails to impact proliferation in triple‐negative breast cancer lines, underscoring its subtype specificity. The clinical relevance of this pathway is reinforced by immunohistochemical analysis showing elevated ARL3 expression in tamoxifen‐resistant recurrent tumors, linking ARL3 levels to treatment failure.

Leveraging structural insights into ARL3's GTPase domain, we identified A‐1331852 as a novel small‐molecule inhibitor capable of disrupting the ARL3‐USP10/ERα axis. This compound demonstrates potent antiproliferative effects in vitro, synergizing with tamoxifen and fulvestrant to overcome endocrine resistance, and suppresses tumor growth in orthotopic xenografts with a favorable safety profile. Mechanistically, A‐1331852‐induced destabilization of ERα and USP10 abrogates both transcriptional oncogenesis and metabolic adaptation, representing a dual attack on HR+ breast cancer's core vulnerabilities. Notably, while recent advances in ERα‐targeting PROTACs have demonstrated potent degradation of ERα and shown promising therapeutic potential, our approach offers a complementary strategy by targeting upstream regulatory nodes that control ERα stability and function, potentially overcoming limitations related to resistance mechanisms or narrow selectivity profiles associated with some direct degraders.^[^ ^45^, ^46^ ^]^ This approach addresses a critical unmet need in endocrine‐resistant disease, where conventional therapies often fail due to ERα dysregulation and metabolic rewiring.^[^ ^37^, ^47^ ^]^

Beyond its established role in ciliary trafficking,^[^ ^25^, ^27^, ^48^ ^]^ our study redefines ARL3 as a noncanonical oncoprotein with pleiotropic functions in HR+ breast cancer. The integration of ERα stabilization, mitophagy regulation, and therapeutic sensitization positions ARL3 as a uniquely promising target for precision medicine. Future studies should explore the clinical utility of A‐1331852 in larger preclinical models, investigate its efficacy in combination with other targeted agents, and characterize its impact on tumor microenvironment interactions. Additionally, exploring ARL3's role in other ERα‐driven diseases, such as endometrial cancer, may expand its therapeutic relevance.

In conclusion, our findings establish a mechanistic framework for ARL3‐dependent oncogenesis in HR+ breast cancer, highlighting its potential as both a biomarker of endocrine resistance and a druggable target for innovative therapeutic strategies. The convergence of transcriptional and metabolic regulation via the ARL3‐ERα axis offers a transformative approach to combating this heterogeneous disease, particularly in patients with treatment‐resistant tumors (Figure 8B).

## 4 Experimental Section

### Clinical Specimens

Matched clinical breast tissue samples (cancerous and adjacent tissues) were procured from BC patients at the First Affiliated Hospital of Chongqing Medical University. All patients were confirmed via pathological biopsy and received surgical resection. ER and PR status were assessed using immunohistochemical analysis. The study was approved by the ethics committee, and patients provided informed consent.

### TCGA and GEO Data Collection

Clinic‐genomic profiles for breast invasive carcinoma, including HTSeq‐FPKM normalized transcriptomics and miRNA data, were retrieved from The Cancer Genome Atlas Program (TCGA). Tamoxifen resistance‐associated mRNA expression datasets (GSE9893, *n* = 155, GSE159968, *n* = 9, GSE125738, *n* = 6, GSE129544, *n* = 2) were obtained from GEO (https://cancergenome.nih.gov). Cross‐platform differential expression analysis identified consensus genes with divergent expression patterns (|log2FC| >1, adj.*p* < 0.05) between tamoxifen‐resistant and sensitive phenotypes.

### Immunohistochemistry (IHC) Staining and Scoring

Immunohistochemical analysis of ARL3, ESR1, and Ki67 was performed on 4 µm FFPE sections from breast cancer specimens (First Affiliated Hospital of CQMU, Ethics Approval K2023‐468) using validated rabbit monoclonal antibodies (ARL3, Proteintech 10961‐1‐AP, 1:200, ESR1, Proteintech 21244‐1‐AP, 1:100, Ki67, Servicebio GB111499‐100 1:500) with citrate‐based antigen retrieval and EnVision detection system. Two blinded pathologists applied a dual‐parameter scoring system (intensity [0–3] × prevalence [0–4], total 0–12) showing strong interobserver agreement (Cohen's κ = 0.82–0.91), evaluating staining intensity (negative to intense) and cellular distribution (<5% to >75% positivity) through standardized semiquantitative assessment.

### Plasmid Construction and Transfection

The lentiCRISPRv2‐sgARL3, lentiCRISPRv2‐sgESR1, lentiCRISPRv2‐sgBCL2, lentiCRISPRv2‐sgBCL2L1, and pLKO.1‐shUSP10 constructs were generated by ligating annealed shRNA oligos into the BsmBI‐linearized lentiCRISPRv2 (Addgene #52961, a gift from Dr. Kang Li) and AgeI/EcoRI‐digested pLKO.1‐RFP vectors, respectively. Target‐specific sequences (validated by Sanger sequencing) are provided in Table S1 (Supporting Information). To establish expression plasmids, full‐length coding sequences of human *ARL3*, wild‐type *ESR1*, and its lysine‐to‐arginine mutants (K252R/K416R) were PCR‐amplified from HEK293T cDNA and subsequently subcloned into the multiple cloning site of the pCDH‐CMV‐EF1α‐CopGFP‐T2A‐Puro lentiviral vector (System Biosciences, CD513B‐1) using In‐Fusion HD cloning. All plasmid transfections were performed in triplicate using Lipofectamine 3000 (Thermo Fisher, L3000015) with optimized DNA/P3000 reagent ratios according to cell confluency.

### Cell Cultures

The human breast cancer (BC) cells derived from ATCC are detailed in Table S2 (Supporting Information). The medium used was high‐glucose Dulbecco's Modified Eagle Medium (DMEM) with pyruvate, which was sourced from Gibco of Thermo Fisher Scientific. It was supplemented with 10% fetal bovine serum (FBS) from ExCell Bio and 1% penicillin/streptomycin from Thermo Fisher Scientific. The cells were placed in an incubator maintained at a temperature of 37 °C and a carbon dioxide (CO_2_) concentration of 5%. All lines were authenticated by 21‐locus STR profiling (>85% matches Cellosaurus v2024_01) and confirmed mycoplasma‐free by PCR within 6 months prior to experimentation.

### RNA Isolation, Reverse‐Transcription Reaction, and Quantitative Real‐Time PCR (qPCR)

Total RNA was isolated from cell monolayers using the Super FastPure Cell RNA Isolation Kit (Vazyme, RC102‐01). First‐strand cDNA synthesis was carried out with 1 µg total RNA using the MCE HiScript IV Reverse Transcriptase (MCE004‐100). Quantitative PCR amplification was conducted in 10‐µL reactions containing SYBR Premix Ex TaqTM II (MedChemExpress) with gene‐specific primers (0.3 µm final concentration, sequences in Table S3, Supporting Information) on a Bio‐Rad CFX96 Touch Deep Well system (Hercules, CA, USA). Experimental groups included three biological replicates with technical duplicates. Expression levels were normalized to Actin endogenous control and quantified via the comparative threshold cycle (2^(−ΔΔCt)) method using CFX Maestro software v2.3 (Bio‐Rad).

### Western Blot Analysis

Cells were lysed under non‐denaturing conditions using ice‐cold RIPA buffer (Beyotime, P0013B) supplemented with 1 × protease inhibitor cocktail (Beyotime, ST506) through 30‐min incubation on a rotary shaker at 4°C. Clarified lysates (20 µg protein/lane, quantified by BCA assay) were resolved on 7.5–15% gradient Tris‐glycine SDS‐PAGE gels under constant voltage (100 V, 90 min) and electrophoretically transferred to pre‐activated PVDF membranes (Merck Millipore, IPFL00010). Membranes were blocked with 5% (w/v) nonfat dried milk (BD Biosciences, 232100) in TBST (pH 7.6) for 1 h at 25 °C before sequential immunoblotting with primary antibodies. Primary antibodies were applied overnight at 4 °C under gentle agitation (20 rpm). Chemiluminescent signals were developed using SuperSignal West Femto Maximum Sensitivity Substrate (Thermo Fisher, 34095) and captured on a ChemiDoc MP Imaging System (Bio‐Rad) with automatic exposure optimization. Band intensity quantification was performed using Image Lab 6.1 software (Bio‐Rad), normalized to β‐tubulin loading controls.

### IF Staining

For immunofluorescence analysis, plasmid‐transfected cells were seeded onto glass coverslips and subjected to methanol fixation 24 h post‐transfection. Primary antibody incubation was performed at 4°C for 12–16 h using the following reagents, anti‐ARL3 (Proteintech 10961‐1‐AP, 1:500), anti‐ESR1 (Servicebio GB151845‐100, 1:500), anti‐USP10 (HUABIO ET1706‐12, 1:100), anti‐LC3B (HUABIO ET1701‐65, 1:100), and anti‐TOMM20 (Proteintech 66777‐1‐Ig, 1:500). Subsequent detection was achieved using species‐matched Alexa Fluor‐conjugated secondary antibodies (Life Technologies A21207/A21202, 1:1000). Nuclear counterstaining was performed with Hoechst 33342 (Sigma–Aldrich) prior to image acquisition using a Nikon A1R confocal microscope (NIS‐Elements software v4.6) with standardized acquisition parameters.

### Cell Viability Assay

Estrogen receptor‐positive breast cancer cell lines (MCF7/T47D) were seeded in 96‐well plates at 3–5 × 10^3^ cells per well under standardized in vitro culture conditions. To evaluate tamoxifen sensitivity, cells were exposed to titrated concentrations of tamoxifen (0–16 µm) for 48 h followed by a 2‐h incubation with CCK‐8 cell viability reagent (TargetMol, C6041). Absorbance values at 450 nm were quantified spectrophotometrically (BioTek Synergy H1) using three independent experimental replicates, with vehicle‐treated controls normalized to 100% viability.

### Colony Formation Assay

Cell growth and colony formation were assessed. In brief, 200 cells were seeded into 12‐well plates and incubated for 14 days to perform the colony formation assay. First, the cells were prefixed with 4% paraformaldehyde at room temperature for 10 min. Subsequently, they were stained with a crystal violet staining solution (Beyotime, #C0121, China) for 30 min at room temperature. After that, the samples were rinsed with distilled water and then photographed under a bright‐field microscope.

### Transwell Migration Assay

Breast cancer cells (MCF7) were resuspended in serum‐free DMEM (Gibco, 11965‐092) at 5 × 10⁴ cells per 200 µL and seeded into the upper chambers of 24‐well Transwell inserts (8 µm pores, NEST, 351152). The lower chambers contained complete medium with 10% FBS as a chemoattractant. After 48 h incubation (5% CO_2_, 37 °C), traversed cells were fixed with 4% paraformaldehyde (Servicebio, G1101), stained with 0.1% crystal violet (Beyotime, C0121), and quantified by counting three random fields per insert using Leica (NIS‐Elements v5.3).

### Wound Healing Migration Analysis

Confluent MCF7 and T47D monolayers (1 × 10⁶ cells per well in six‐well plates) were mechanically wounded using sterile 10 µL pipette tips. Following PBS washing (× 3) to remove debris, cultures were maintained in 2% FBS medium (ExCell Bio) to minimize proliferation interference. Phase‐contrast images were acquired at 0/24/48 h intervals using a Leica DMi8 inverted microscope (10 × objective) equipped with an on‐stage incubator (37°C/5% CO_2_).

### Animal Models

All animal studies were performed in accordance with ARRIVE criteria under ethical approval from Chongqing Medical University IACUC (Approval ID, K2023‐468). Female BALB/c nude mice (4‐week‐old) were housed in specific pathogen‐free (SPF) conditions with controlled photoperiod (12‐h light/12‐h dark), temperature (22–26°C), and humidity (50–70%).

For tumor initiation, estradiol cypionate (30 µg dissolved in 30 µL corn oil, Beyotime) was delivered intramuscularly 7 days before cell engraftment. In the proliferation assay, suspensions containing MCF7‐sgNC or ARL3‐KO cells (5 × 10⁶ cells per mouse in 1:1 PBS/Matrigel mixture, BD Biosciences) were surgically transplanted into the fourth mammary fat pads, with tumor development monitored over a 42‐day period.

For drug responsiveness evaluation, when xenografts reached 100 mm^3^ (width^2^ × length/2), animals were randomly assigned to receive either vehicle control or tamoxifen (250 mg kg^−1^ week^−1^ via subcutaneous injection, TargetMol #10540‐29‐1) for 28 days (*n* = 5 per cohort).

In combination therapy trials, MCF7‐sgNC(5 × 10⁶ cells per mouse in 1:1 PBS/Matrigel mixture, BD Biosciences) were surgically transplanted into the fourth mammary fat pads, mice bearing 100 mm^3^ tumors were stratified using Microsoft Excel RAND algorithm into four treatment arms, vehicle control, tamoxifen monotherapy (250 mg/kg/week, SC), A‐1331852 monotherapy (25 mg^−1^ kg^−1^ day^−1^ oral administration, TargetMol #1430844‐80‐6) and tamoxifen + A‐1331852 (14‐day oral A‐1331852 with concurrent 4‐week tamoxifen). The dosage of A‐1331852(25 mg kg^−1^)was based on a previous study.^[^ ^49^, ^50^ ^]^

Outcome assessments were conducted by personnel blinded to experimental groupings.

Terminal procedures involved euthanasia via cervical dislocation under isoflurane anesthesia, followed by the collection of tumors and visceral organs (heart, liver, spleen, lungs, kidneys). Tissues were weighed, imaged (Leica DMi8), formalin‐fixed, and processed into paraffin sections for immunohistochemical (IHC) characterization.

### RNA‐Seq

RNA‐seq was performed on CRISPR‐engineered MCF7 cells (sgNC vs ARL3‐KO) with triplicate biological replicates cultured in standardized 10% FBS/DMEM‐F12. Total RNA was isolated using TRIzol (Invitrogen) with DNase I treatment, and RNA integrity was verified (RIN≥8.0, Agilent 2100 Bioanalyzer). Strand‐specific libraries prepared with the NEBNext Ultra II kit (2 µg RNA per sample) were sequenced on Illumina NovaSeq 6000 (150PE, >40M reads/sample).

Raw reads processed by fastp (Phred≥20) were aligned to GRCh38 using Rsubread, with gene quantification via featureCounts. DESeq2 identified DEGs (FDR<0.05, |log2FC|>1), visualized by pheatmap. Functional enrichment combined GO/KEGG over‐representation analysis and MSigDB‐based GSEA (clusterProfiler).

### Co‐IP and LC‐MS/MS

Cellular lysates were prepared in ice‐cold IP lysis buffer (20 mm Tris‐HCl pH 7.5, 150 mm NaCl, 1% NP‐40, Beyotime P0013) supplemented with EDTA‐free protease inhibitor cocktail (Roche cOmplete, 1 tablet/10 mL). For reciprocal immunoprecipitation, precleared lysates (14 000 × g, 15 min at 4°C) were incubated with anti‐HA magnetic beads (MedChemExpress HY‐K0201, 50 µL beads mg^−1^ protein), anti‐FLAG magnetic beads (MedChemExpress HY‐K0207, 50 µL beads mg^−1^ protein), or species‐matched IgG control (Beyotime A7016) under gentle rotation (16 h, 4°C). Protein complexes were captured using Protein A/G magnetic beads (MedChemExpress HY‐K0202) pre‐blocked with 5% BSA/PBS, followed by five stringent washes with high‐salt buffer (500 mm NaCl, 0.1% Tween‐20). Eluted proteins (0.1 m glycine pH 2.5) were neutralized with 1 m Tris‐HCl pH 8.0, and subjected to Immunoblotting using precast 4–20% gradient gels (Bio‐Rad #4561094) with ECL Prime detection (Biosharp).

LC‐MS/MS analysis was conducted by Cosmos Wisdom (Hangzhou, China). The peptide samples were dissolved in mobile phase A (0.1% formic acid) and separated by EASY nLC‐1200 (Thermo Scientific). The isolated peptides were subjected to Nano source followed by a Q Exactive HF‐X mass spectrometer.

### Seahorse XF Mitochondrial Stress Assay

For mitochondrial function assessment using the Seahorse XF platform, cells were plated in XFe96 microplates at 4 × 10⁴ cells per well and cultured for 24 h prior to analysis. The mitochondrial stress test was performed according to manufacturer protocols (Agilent Technologies) with an XFe96 Analyzer. Mitochondrial parameters were measured in XF base medium containing glucose, pyruvate, and glutamine (pH 7.4) through sequential administration of metabolic modulators, 1.5 µm oligomycin (ATP synthase inhibitor), 1.5 µm FCCP (mitochondrial uncoupler), followed by 0.5 µm rotenone/antimycin A (complex I/III inhibitors). Real‐time oxygen consumption rate (OCR) measurements were recorded following each compound injection.

### Cellular ATP Content Analysis

Cellular ATP quantification was performed using the Enhanced ATP Assay Kit (Beyotime Biotechnology, S0027) with protocol modifications. After removing culture medium, cells in 6‐well plates were lysed with 200 µL per well ice‐cold lysis buffer, followed by centrifugation (12 000 g, 5 min, 4°C) to collect clarified supernatants. Simultaneously, the ATP detection reagent was diluted 1:4 (v/v) with the manufacturer‐provided diluent to prepare a working solution. For bioluminescent analysis, 100 µL of working solution was pre‐equilibrated in assay tubes (3–5 min, RT) to stabilize baseline signals. Subsequently, 20 µL of supernatant was added to the tubes, immediately vortex‐mixed for 10 s, and relative light units (RLU) were recorded using a luminometer. Protein concentrations were normalized across samples prior to ATP measurement via parallel BCA quantification of lysate aliquots.

### Transmission Electron Microscopy (TEM)

For TEM sample preparation, centrifuge cells/bacteria (min. vol. ≈0.5–1 mm^3^), fix in glutaraldehyde at 4 °C for 2–4 h, and store in fixative. Wash pellets with 0.1 m PB (pH 7.4), embed in 1% agarose, and solidify at 4 °C. Postfix with 1% osmium tetroxide in PB (pH 7.4) for 2 h in the dark at RT, rinse with PB. Dehydrate in ethanol gradient (30%–100%, 20 min step^−1^), then acetone. Infiltrate with EPON 812 resin‐acetone mixtures (1:1, 37°C, 2–4 h, 1:2, 37°C, overnight) and pure resin (37°C, 5–8 h), embed, and polymerize at 37°C overnight. Cure at 60°C for 48 h, cut 60–80 nm sections, and collect on Formvar grids. Stain with uranyl acetate and lead citrate, air‐dry, and image at 80 kV.

### Virtual Screening

The computational workflow commenced with de novo protein structure prediction using AlphaFold2, leveraging the target's amino acid sequence as input. Following protonation‐optimized ligand library preparation, hierarchical virtual screening was conducted through iterative molecular docking employing hybrid scoring functions (Vina‐XGB and Glide SP/XP). Candidate prioritization integrated dual criteria: 1) superior docking scores within the top 5th percentile and 2) MM/GBSA‐derived binding energies below −30 kcal mol^−1^ threshold, ensuring selection of optimal ARL3‐targeting compounds. The procedures were performed by APExBIO (Shanghai, China).

### Molecular Dynamics (MD) Simulations

Molecular dynamics simulations were performed using Amber24 with the ff19SB force field ^[^ ^31^, ^51^ ^]^ and the OPC water model.^[^ ^52^, ^54^ ^]^ The protein‐ligand complex was solvated in a cubic periodic box, with electrostatic/van der Waals cutoffs set to 1.0 nm and long‐range electrostatics handled by PME.^[^ ^53^ ^]^ After energy minimization (5000 steps), the system underwent sequential equilibration under NVE (200 ps) and NPT (100 ps) ensembles at 300 K/1 bar controlled by V‐rescale and Parrinello‐Rahman methods, followed by 100 ns production MD with a 2‐fs timestep. Trajectory analyses, including RMSD, RMSF, radius of gyration, and hydrogen bonds, were computed using cpptraj and custom Python scripts.

### MicroScale Thermophoresis (MST) Analysis

The binding affinity between the ligand and target protein was determined using MicroScale Thermophoresis (MST). The ligand was serially diluted to concentrations ranging from 10–8 to 10–4M. MST measurements were conducted using a Monolith NT.115 instrument at 25 °C with 20% LED and 40% infrared laser power. The normalized fluorescence intensity (Fnorm) was recorded and analyzed using MO. Affinity Analysis software to generate a dose‐response curve and determine the dissociation constant (Kd).

### Cellular Thermal Shift Assay (CETSA)

Cellular Thermal Shift Assay was conducted to evaluate the target engagement of A‐1331852 (1 µm, 4‐h treatment) with ARL3. Treated cells were heated to a gradient of temperatures (46–56°C), lysed, and centrifuged. The remaining soluble ARL3 in the supernatants was then quantified by western blotting.

### Statistical Analysis

Statistical analyses were conducted in GraphPad Prism 9 (RRID:SCR_002798) with the following methodology: for multigroup comparisons, one‐way ANOVA was employed, while two‐group comparisons utilized unpaired two‐tailed Student's *t*‐tests. Specific paired analyses (e.g., tumor vs adjacent normal tissues) were applied to paired Student's *t*‐tests. Statistical significance was defined as *p* < 0.05 across all comparisons. Experimental reproducibility was ensured through triplicate biological replicates (n ≥ 3).

### Ethics Statement

All experimental protocols involving human participants and biological specimens were conducted in strict accordance with the ethical principles outlined in the Declaration of Helsinki. The Institutional Review Board of The First Affiliated Hospital of Chongqing Medical University independently reviewed and authorized this study (Approval Code, K2023‐468, Date, October 11, 2023), confirming full compliance with international ethical standards and informed consent procedures.

## Conflict of Interest

The authors declare no conflict of interest.

## Author Contributions

H.L., Y.L., and Z.H.C. contributed equally to this work as co‐authors. Y.L., S.G., Z.H.C. and K.H. conceived the study and designed the experiments. H.L., Y.L., and Z.H.C. carried out most of the experiments and analyzed the data. K.L., A.L.L., D.S., X.L., Y.P. helped with the experiments and provided technical assistance. K.L. and D.S. provided equipment. S.P.G., H.C.Y., A.S.J., M.Y.S., and S.C.L. provided funding support. S.C.L. supervised the project, coordinated the research team, and oversaw the research direction and experimental design. H.L., M.Y.S., and S.C.L. wrote and revised the manuscript. All authors reviewed and approved the final manuscript.

## Supporting information



Supporting Information

## Data Availability

The TCGA repository (https://cancergenome.nih.gov/) and GEO repository (https://www.ncbi.nlm.nih.gov/geo/) contains the datasets that were evaluated for this work. The article and Supplementary Materials contain the original contributions that were made during the investigation (Figure S7, Supporting Information). Any additional questions can be directed to the corresponding author.
