# Supplementary material for: ARL3 Enhances ERα Stability via USP10 Deubiquitination to Promote Endocrine Resistance and Drive Mitochondrial Metabolic Reprogramming in HR+ Breast Cancer
Source: Adv Sci (Weinh). 2025 Oct 5;12(47):e09769. doi: 10.1002/advs.202509769 (PMC12713097; doi:10.1002/advs.202509769)
Supplement: Supplementary file 1 — Supporting Information [file ADVS-12-e09769-s001.docx]

Supplementary Table 1. sgRNA and shRNA target sequences.

| *Gene* |  | Target sequence |
| --- | --- | --- |
| *ARL3* | sgRNA | CTGAATGTATGGGACATTGG |
| *USP10* | shRNA | GCCTCTCTTTAGTGGCTCTTT |
| *ESR1* | sgRNA | CTGACCGTAGACCTGCGCGT |
| *BCL2* | sgRNA | CTGACGCCCTTCACCGCGCG |
| *BCL2L1* | sgRNA | CGCTTGCTTTACTGCTGCCA |

Supplementary Table 2. Cell sources.

| *Cell line* | Source | RRID |
| --- | --- | --- |
| *MCF7* | ATCC HTB-22 | CVCL_0031 |
| *T47D* | ATCC HTB-133 | CVCL_0553 |
| *SKBR3* | ATCC HTB-30 | CVCL_0033 |
| *BT474* | ATCC HTB-20 | CVCL_0179 |
| *BT-549* | ATCC HTB-122 | CVCL_1092 |
| *MDA-MB-436* | ATCC HTB-130 | CVCL_0623 |
| *MDA-MB-231* | ATCC HTB-26 | CVCL_0062 |
| *MDA-MB-468* | ATCC HTB-132 | CVCL_0419 |

Supplementary Table 3. Qpcr primers.

| Gene | Qpcr primers |
| --- | --- |
| *ARL3-F* | TACTTCTCCTGGGCTTGG |
| *ARL3-R* | CCAGTAATTCCGCTAGTTCC |
| *ESR1-F* | ATGGAGTCTGGTCCTGTG |
| *ESR1-R* | CTGTTCTTCTTAGAGCGTTT |
| *GREB1-F* | GACCATCGGCTTTAGG |
| *GREB1-R* | TACTGCTCGTATGCCCGTGA |
| *MYC-F* | ACACCCTTCTCCCTTCG |
| *MYC-R* | GTGGCCGCGGGCGGGGTTCG |
| *PTGS2-F* | CATCTACACGCCATTCA |
| *PTGS2-R* | ATAGGTGAAGAGGAACAGCA |
| *SERPINE1-F* | CTGGTGAATGCCCTCT |
| *SERPINE1-R* | CAGGAGGCGGGGCAGCCTGG |
| *USP10-F* | CTATTTGAAAGATGGTGGCG |
| *USP10-R* | CAGCAGGAGGTGATGCACTC |
